# Human Sensation of Transcranial Electric Stimulation

**DOI:** 10.1038/s41598-019-51792-8

**Published:** 2019-10-24

**Authors:** Fan-Gang Zeng, Phillip Tran, Matthew Richardson, Shuping Sun, Yuchen Xu

**Affiliations:** 1 Center for Hearing Research, Departments of Anatomy and Neurobiology, Biomedical Engineering, Cognitive Sciences, Otolaryngology – Head and Neck Surgery, University of California Irvine, Irvine, California 92697 USA; 2 Department of Otolaryngology – Head and Neck Surgery, The First Affiliated Hospital, Zhengzhou University, Henan, 450052 China; 3 Department of Precision Instrument, Tsinghua University, Beijing, 100084 China

**Keywords:** Sensory processing, Translational research

## Abstract

Noninvasive transcranial electric stimulation is increasingly being used as an advantageous therapy alternative that may activate deep tissues while avoiding drug side-effects. However, not only is there limited evidence for activation of deep tissues by transcranial electric stimulation, its evoked human sensation is understudied and often dismissed as a placebo or secondary effect. By systematically characterizing the human sensation evoked by transcranial alternating-current stimulation, we observed not only stimulus frequency and electrode position dependencies specific for auditory and visual sensation but also a broader presence of somatic sensation ranging from touch and vibration to pain and pressure. We found generally monotonic input-output functions at suprathreshold levels, and often multiple types of sensation occurring simultaneously in response to the same electric stimulation. We further used a recording circuit embedded in a cochlear implant to directly and objectively measure the amount of transcranial electric stimulation reaching the auditory nerve, a deep intercranial target located in the densest bone of the skull. We found an optimal configuration using an ear canal electrode and low-frequency (<300 Hz) sinusoids that delivered maximally ~1% of the transcranial current to the auditory nerve, which was sufficient to produce sound sensation even in deafened ears. Our results suggest that frequency resonance due to neuronal intrinsic electric properties need to be explored for targeted deep brain stimulation and novel brain-computer interfaces.

## Introduction

Human sensation of electric stimulation was first reported in the 1800s, when Alessandro Volta invented the battery and studied its electrophysiological effects on his own body^1^. By placing two ends of a battery (with a potential difference as high as 50 volts) in his eye, ear, mouth, nose, and skin, Volta described the sensations of seeing light, hearing noise, tasting acid, and feeling pain. Most importantly, Volta’s experimentation revealed a cardinal principle of the functional organization of the nervous system: that sensation depends on the type of nerve stimulated rather than the type of stimulus used^2^.

With advances in electronics^3^ in the mid 1900s, human sensations to electric stimulation received renewed attention and served as a tool for understanding structure-function mechanisms. For instance, Motokawa^4^ and Howarth^5^ showed robust electrically-evoked visual sensations (called phosphenes or flickers) that appeared independently of stimulus waveforms, electrode types, and positions in the head. Brindley ^6^ later determined the site of excitation to be either photoreceptors or bipolar cells in the retina. Other studies placed electrodes near the ear or in the ear canal, and reported auditory sensations in response to alternating-current electric stimulation^7–9^. Jones *et al*.^10^ and Flottorp ^11^ identified at least three mechanisms underlying this type of electric hearing, including direct conversion of electric currents into mechanical vibrations, activation of cochlear hair cells, and activation of the auditory nerve. Finally, Bishop ^12^ elucidated various types of electrically-evoked somatic sensation (e.g., itches, touch, pricks, or pains) at the single receptor level by applying a needle electrode while avoiding mechanical deformations of skin. Bypassing somatic receptors to electrically stimulate the sural nerve in patients who received cordotomy, Collins *et al*.^13^ later established a causal relationship between various types of the sural nerve and their separate contributions to touch, temperature and pain sensation.

Following these initial electric sensation studies, general interest shifted towards more translational research. On the one hand, invasive electric stimulation was developed to directly activate the peripheral or central nerves for restoring impaired sensory or motor functions^14–16^. On the other hand, non-invasive transcranial technologies using different waveforms with single or multiple electrode pairs^17,18^ have been developed to not only treat neurological disorders^19–22^ or augment rehabilitation^23,24^ but also enhance normal functions in healthy individuals^25–27^. At present, transcranial electric stimulation is considered as an advantageous therapy alternative because it does not require surgery, has minimal adverse effects and may activate neural tissues to affect brain network dynamics and behavior^28–30^. However, a systematic study on human sensation of transcranial electric stimulation is still lacking with existing studies focusing on sensation in one or two modalities^31–35^, and treating these sensations as factors that may break the blinding of the participants and contribute to the development of placebo effects^36–40^. Moreover, direct evidence is limited for deep neural activation by transcranial electric stimulation^41–43^.

Because sensory receptors and neurons have different locations and electric properties, we hypothesized that transcranial electric stimulation produces different types of sensation, depending on both electrode position and stimulus parameters. This hypothesis led to the two main goals of the present study. The first goal was to systematically characterize human sensations to transcranial electric stimulation. On three different electrode types and eight different electrode positions, we varied the frequency and level of alternating-current sinusoids to measure each sensation’s threshold and input-output functions. These functions were obtained using standard psychophysical methods in adult human subjects who sat in a dark, double-walled sound-proof booth. The second goal was to empirically determine whether transcranial stimulation can actually activate the auditory nerve, a deep target that not only resides in the densest bone of the skull, but also is only 3–4 cm from the subthalamic nuclei, a deep brain stimulation target for tremor suppression^44^. To achieve the second goal, we used two novel techniques, including a gold-plated tip electrode that can be reliably placed in the ear canal and an embedded recording circuit^45^ in a cochlear implant that can directly measure the amount of electric current reaching the auditory nerve from transcranial electric stimulation.

## Results

**Figure 1:**
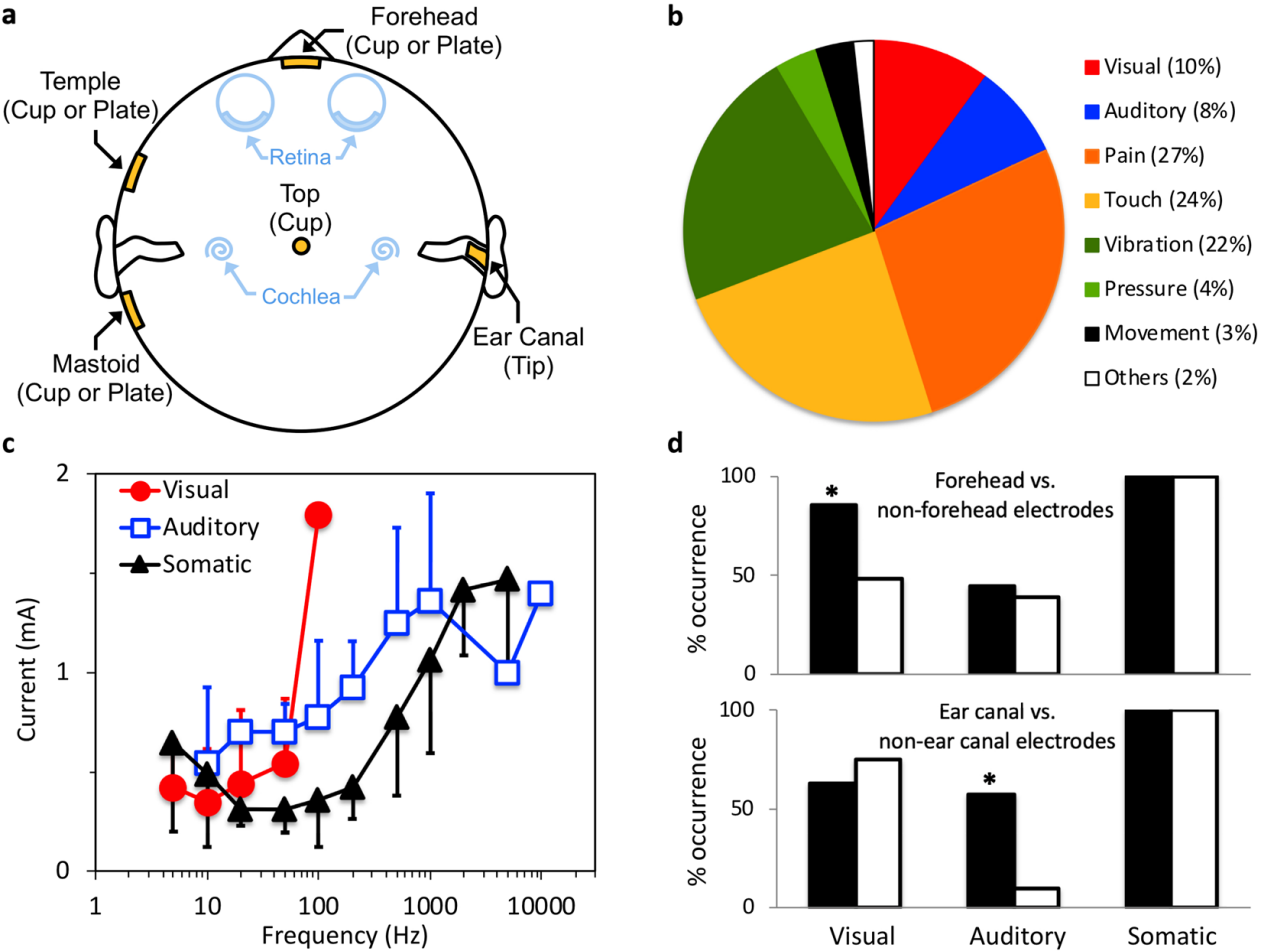
Human sensation evoked by transcranial electric stimulation. The human subject was tested in a double-walled, sound-proof booth, with the light turned off. All stimuli were charge-balanced and delivered through a current source. The sensation threshold was obtained by the method of limits. **(a)** Electrode montages consisted of eight positions: forehead, top of the head, left and right temple, left and right mastoid, and left and right ear canal (note: only the left temple, left mastoid, and right ear canal electrodes are displayed in the diagram in yellow). The forehead and ear canal electrodes were located closest to the visual and auditory organs: the retina and cochlea, respectively (blue). **(b)** Distribution of 983 reported sensations by type and other neural and muscular activities (see text for details). **(c)** Visual, auditory, and somatic sensitivity thresholds in electric current are plotted as a function of stimulus frequency (5 to 10,000 Hz) averaged over all electrode positions. Error bars represent one standard deviation of the mean across all electrode positions. **(d)** Percentage (y-axis) for each of the three types of sensation (regardless of stimulus frequency and level) for two subsets of electrode configurations: those containing forehead electrode (filled bars in the top panel) vs. non-forehead electrodes (open bars); those containing ear canal electrodes (filled bars in the bottom panel) vs. non-ear-canal electrodes (open bars). The asterisk (*) indicates a significant difference between two occurrences [two-tailed binomial distribution test: *z(n*_1_* = *27, *n*_2_* = *33) = 2.97, p = 0.003 for the visual sensation between forehead and non-forehead electrodes; *z(n*_1_* = *38, *n*_2_* = *20) = 3.49, p = 0.0005 for the auditory sensation between ear canal and non-ear canal electrodes. See Procedures in Methods].

The presented results are based on ~88,000 independent reports made by 20 adult subjects using 8 electrode positions (Fig. 1a, yellow symbols) and 3 electrode types: a plate, a cup or a tip electrode (see Methods). Six of the eight electrode positions were designed to cover the front, side and top planes of the scalp, while the other 2 tip electrodes were inserted in the ear canal to allow close and non-invasive stimulation of the cochlea. The experiments involved presenting a sinusoidal stimulus (with different frequencies ranging from 5 to 10,000 Hz and current levels up to 2 mA peak amplitude) to safely deliver charge-balanced stimulation to the subjects, and to systematically characterize evoked sensations as a function of stimulus frequency and intensity. Under these electrode and stimulus conditions, the subjects produced a total of 983 positive reports on a variety of sensations and occasionally sensorimotor activities (Fig. 1b). Of the 983 reports, visual sensation was reported 10% of the time as white flickers with various brightness and occupying different regions and sizes in the visual field. Auditory sensation was reported 8% of the time as sounds with either tonal or noisy qualities. Somatic sensation was reported 77% of the time as feelings of pain (27%), touch (24%), vibration (22%), and pressure (4%). The pain sensation was described as a prick or a sting, while the touch sensation was described as a tingle or a tap. Motor activities were reported 3% of the time, including visible facial movements of the jaw, neck, pinna or eyebrows. The jaw, neck and pinna movements were evoked by 10–1000 Hz stimulation using the ear canal and mastoid configuration, whereas the eyebrow movement was caused only by 10-Hz forehead and mastoid stimulation. The remaining 2% of reports included dizziness, hotness or numbness, where dizziness was evoked by either ear canal or mastoid electrodes and hotness or numbness could be evoked by any electrode configurations. No taste, smell or any other sensation was reported.

### Dependence on stimulus frequency and electrode position

We observed that electrically evoked sensation depends on stimulus frequency (Fig. 1c). Regardless of electrode positions, for current levels up to 2 mA over the sampled frequencies from 5 to 10,000 Hz, only low frequencies between 5 and 100 Hz evoked a visual sensation, with a minimum threshold of 0.35 mA at 10 Hz. Although the 10-Hz electric stimulation also produced the lowest threshold of 0.54 mA for auditory sensation, the auditory responsive frequency range (10–10,000 Hz) was 100 times wider than the visual frequency range (5–100 Hz). Somatic sensation had a responsive frequency range (5–5,000 Hz) as wide as the auditory range, and the lowest overall threshold (0.31 mA) at 50 Hz.

We also observed that electrically evoked sensation depends on electrode position (Fig. 1d). Regardless of stimulus frequency and level, the subset of configurations containing forehead electrodes (filled bars in the top panel) were nearly twice as likely to evoke visual sensation than those containing non-forehead electrodes (open bars, 85% vs. 48%). Forehead or non-forehead electrodes produced no significant difference in evoking either auditory (~40%) or somatic (100%) sensation. Given the geometric proximity to the auditory organ, an ear canal electrode (filled bars in the bottom panel) was 6 times more likely to evoke auditory sensation than no electrodes in the ear canal (open bars, 58% vs. 10%). The presence or absence of an ear canal electrode did not produce any difference in evoking visual (~70%) or somatic (100%) sensation. Finally, none of the other electrode positions, namely, top of the head, mastoid, and temple, produced any difference in evoking visual, auditory or somatic sensation.

### Individual input-output functions

**Figure 2:**
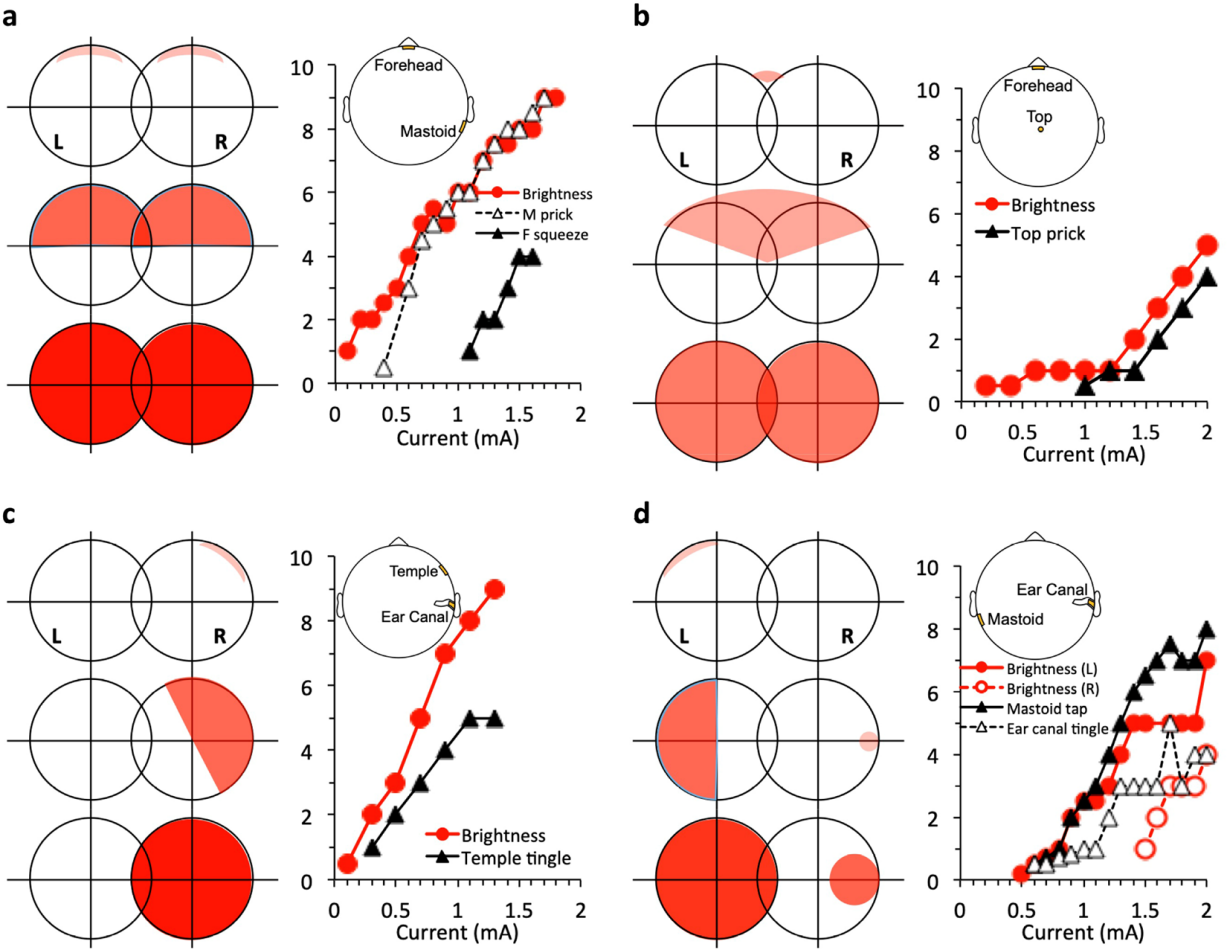
Left and right fields (L and R circles) of visual sensation and magnitude estimates as a function of electric current in mA (line-scatter charts). The top, middle, and bottom visual fields indicate threshold, mid, and upper level sensations. All stimuli were at 10 Hz. **(a)** Forehead and right mastoid plate electrodes. **(b)** Forehead plate electrode and top (Cz) cup electrode. **(c)** Right ear canal tiptrode and right temple plate electrode. **(d)** Right ear canal tiptrode and left mastoid plate electrode.

We observed typically symmetrical visual sensation with forehead electrodes but asymmetrical visual sensation with non-forehead electrodes in response to 10-Hz sinusoidal electric stimulation (Fig. 2). The forehead and right mastoid plate electrode combination produced one dim white flicker on top of both visual fields (top two drawings of the left panel in Fig. 2a) with a brightness estimate of 1 out of 10 at 0.1 mA (right panel in Fig. 2a). The visual sensation increased to two brighter white flickers covering the top half of the visual fields at 0.9 mA (middle two drawings of the left panel in Fig. 2a) and eventually three extremely bright white flickers saturating the entire visual field at 1.8 mA (bottom two drawings of the left panel in Fig. 2a). The visual sensation was also accompanied by two types of somatic sensation, including monotonically increasing pressure (“squeeze” = solid triangles in the right panel in Fig. 2a) on the forehead electrode and pain (“prick” = open triangles in right panel in Fig. 2a) on the mastoid electrode. The forehead and top electrode montage produced a faint white flicker near the top of the visual field at a 0.2 mA threshold level, spreading towards lower and peripheral visual fields until the entire visual field was occupied at the 2-mA level (Fig. 2b). The monotonic brightness function was accompanied by a similar magnitude growth function of somatic sensation described as a “prick” on the top of the head. In contrast to the symmetric sensation described thus far, the right ear canal and right temple electrodes (Fig. 2c) produced an asymmetric visual sensation, starting with a dim white flicker in the top-peripheral corner of the right visual field and then transitioning to two bright white flickers covering the entire right field. The visual sensation was also accompanied by a “tingle” on the right temple. The right ear canal and left mastoid electrodes (Fig. 2d) also produced an asymmetric visual sensation, with a dim flicker in the top-peripheral corner of the right visual field and a dim circle on the right side, which increased to two brighter flickers covering the entire left field and part of the right field, respectively, with increasing current. The accompanying somatic sensation included a “tingle” in the right ear canal and a “tap” on the left mastoid. The electric dynamic range, defined as 20 log (*I*_*m*_/*I*_*θ*_), where *I*_*m*_ is the current level evoking the maximal sensation and *I*_*θ*_ is the threshold level, was 17 ± 8 dB (STD = standard deviation across individual subjects) for visual sensation and 10 ± 3 dB for somatic sensation.

**Figure 3:**
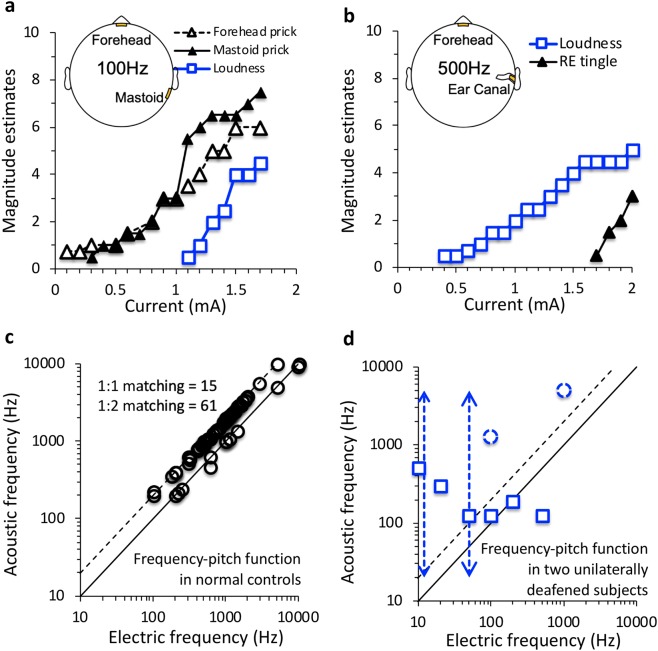
(**a**) Magnitude estimates as a function of electric current for right mastoid and forehead plate electrodes in an individual subject (blue = auditory sensation; black = somatic sensation). **(b)** Same as (**a**) except for right ear canal tiptrode and forehead plate electrode. (**c**) Frequency-pitch function from 14 normal-hearing subjects. The solid diagonal line represents 1:1 matches between acoustic frequency (y-axis) and electric frequency (x-axis). The dashed line represents 2:1 matches, with acoustic frequency doubling electric frequency. **(d)** Frequency-pitch function in 2 unilaterally deafened subjects (dashed lines and circles for one subject and solid squares for the other). The y-axis represents sound frequencies in the hearing ear matched to the frequency of electric stimulation delivered to the deaf ear (x-axis). Dashed lines = subject U1, Solid lines = subject U2 (See Subjects in Methods).

Transcranial sinusoidal electric stimulation produced monotonically-increasing loudness growth, which was always accompanied by somatic sensation. One typical case showed that electric stimulation using forehead and right mastoid plate electrodes produced not only auditory sensation (blue squares in Fig. 3a), but also pain sensation at both electrode sites (black triangles in Fig. 3a). The pain sensation was observed either at low current levels when no auditory sensation was reported (0.1–1 mA) or was rated to have greater magnitude than the auditory sensation at higher levels (1.1–1.8 mA) when both types of sensation were simultaneously present. The other representative case involved the use of an ear canal electrode, which usually produced more dominant auditory sensation (blue squares in Fig. 3b) than somatic sensation (black triangles in Fig. 3b). The electric dynamic range across 14 subjects was 12 ± 7 dB for auditory sensation and 14 ± 8 dB for somatic sensation. The somatic dynamic range combined from both visual and auditory measures was 12 ± 6 dB. These normal-hearing subjects reported auditory sensation as either tonal or harmonic in quality. Out of 76 reports, 80% of the subjects matched the perceived pitch of electric stimulation to an acoustic frequency that was an octave higher than the electric frequency (the dashed line in Fig. 3c). For example, a 500-Hz electric stimulus sounded like a 1000-Hz pure tone. The observed mean ratio between acoustic and electric frequency was 1.97 ± 0.07 (STD). The remaining 20% matched the electric frequency to the same acoustic frequency, with the mean ratio being 0.99 ± 0.06 (the solid line in Fig. 3c).

### Auditory nerve activation

We observed direct activation of the auditory nerve by transcranial electric stimulation in two unilaterally deafened subjects. With tiptrodes placed in both ear canals, the two subjects heard sounds in their deafened ears. In contrast from the control subjects who perceived sounds of tonal quality, these two subjects perceived the sound as “a rattle-snake noise”, “buzzing”, “pinging”, or “chirping”. One subject matched 10- or 50-Hz electric stimulation to a 20–5,000 Hz broadband noise in the hearing ear (dashed lines with arrows in Fig. 3d), and other electric stimuli to tones that had much higher frequencies (dashed circles in Fig. 3d) than the normal frequency range (solid or dashed diagonal line in Fig. 3d). The other subject matched 50–200 Hz electric stimulation to the normal frequency range, but 10- and 20-Hz to much higher frequencies while 500-Hz to a much lower frequency (squares in Fig. 3d). The average dynamic range from both subjects was 14 ± 7 dB, not significantly different from the 12 ± 7 dB control range (one-tail t-test, p = 0.34).

We recorded the amount of current flowing into the cochlea from transcranial electric stimulation using the cochlear implant built-in telemetry in the second subject^46^. The back-telemetry uses the stimulating electrodes as recording electrodes in the cochlea and sends the recorded electrical or neural signals from the implant to the outside unit^45,47^. The back-telemetry recording showed that the ipsilateral and contralateral ear canal electrode configuration produced the largest gain of 0.84%, while the ipsilateral mastoid and temple electrode configuration produced the lowest gain of 0.07%. The back-telemetry result suggested that although 99% of transcranial electric stimulation dissipated into tissues between the scalp and the cochlea, the remaining 1% of a 2-mA current, or up to 20 μA, that reached inside the cochlea was sufficient to activate the auditory nerve in the present cases.

## Discussion

The present study characterized the human sensation of transcranial electric stimulation over a wide range of stimulus parameters and electrode montages. Consistent with our working hypothesis, we found that the visual sensation occurred preferentially with low-frequency (5–100 Hz) stimulation using the forehead electrode, the auditory sensation with broad-frequency (10–10,000 Hz) stimulation using the ear canal electrode, whereas the tactile sensation at all frequencies with all electrodes. In addition, we found generally monotonic sensation magnitude growth as a function of stimulus level and frequently multiple sensations occurring simultaneously in response to the same electric stimulation. Here we compare our results with previous electric sensation studies and normal sensation caused by naturally occurring stimuli, while discussing mechanisms and implications of human sensation evoked by transcranial electric stimulation.

### Comparison with previous studies and normal sensation

**Figure 4:**
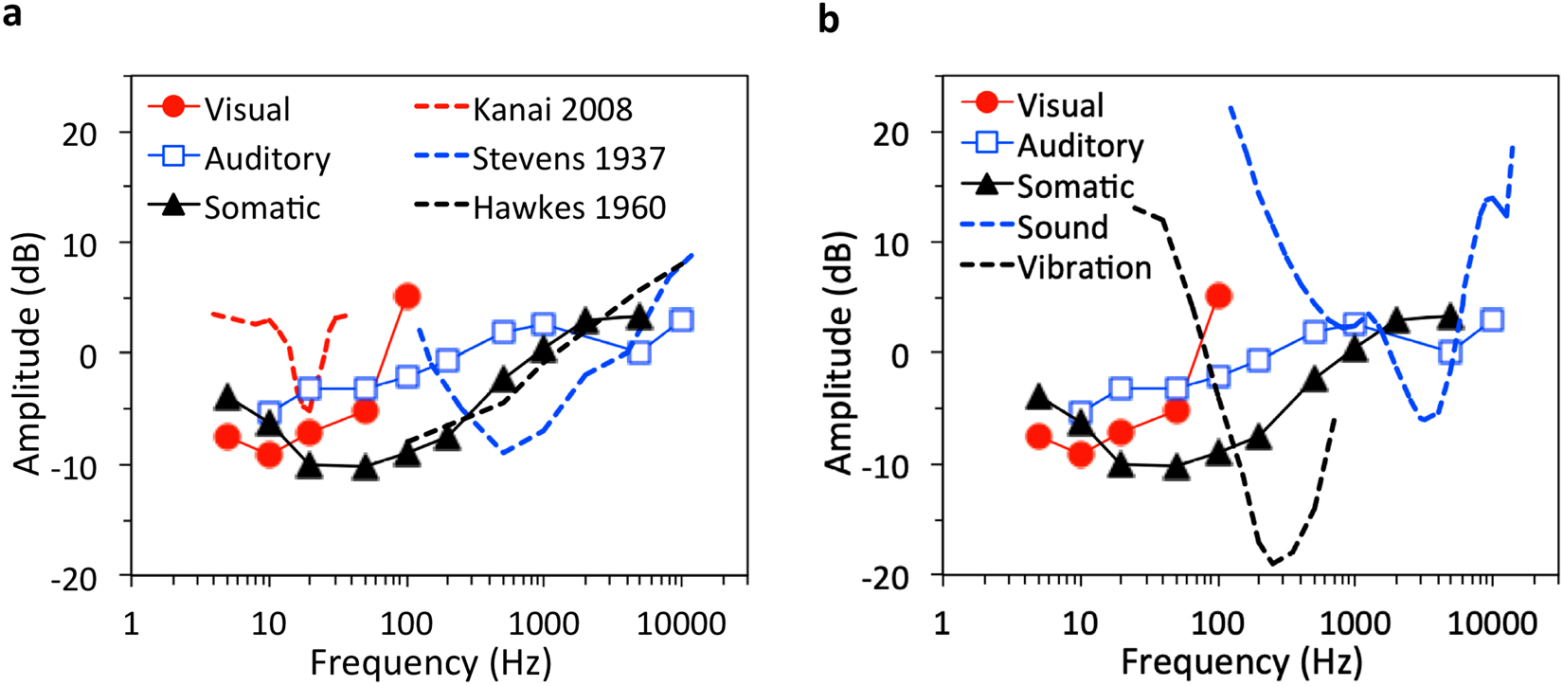
Electric and normal sensation thresholds (**a**) Comparison of the present result (symbols) with previous studies (dashed lines). The Kanai et al. study used two rubber electrodes with one being placed on the back of the head (inion, 3 × 4 cm) and another on the vertex (6 × 9 cm)^32^. The Stevens study used a copper wire immersed in saline solution that filled the ear canal and an aluminum disk strapped on the forearm^8^. The Hawkes study used a ring electrode on the finger and a round electrode on the palm^48^. All amplitude data were converted into dB referring to 1 mA except for electric hearing data from Stevens 1937, in which dB values were referred to 10 μW. **(b)** Comparison with normal auditory sensation thresholds (dB RE: 20 μPa) evoked by sound^98^ and normal tactile sensation thresholds evoked by vibration (dB RE: 1 μm)^99^.

We replicated the low-frequency dependency of electrically-evoked visual sensation, but our data (red circles in Fig. 4a) showed lower thresholds and broader frequency selectivity than the previous study (dashed red line in Fig. 4a)^32^. These two differences were possibly due to the different electrode positions between the two studies, with the previous study placing the active electrode on the back of the head^32^. Our auditory sensation range (blue squares) was  similar to the previous study (blue dashed line)^8^ between 100 and 10,000 Hz, however, our data showed maximal sensitivity at 10 Hz compared to 250 Hz from the previous study. Our study (black triangles) and a previous study (black dashed line)^48^ matched in threshold between 100 and 10,000 Hz for somatic sensations, with our study extending the lower boundary to 10 Hz and showing maximal sensitivity at 20 Hz.

It is also interesting to compare the electrically evoked sensation with normal sensation caused by sound and vibration (Fig. 4b). First, both electric and acoustic stimuli evoked auditory sensations between 100 and 10,000 Hz, but electric stimulation is most sensitive from 10 to 100 Hz while acoustic stimulation is most sensitive between 2,000 and 4,000 Hz (dashed blue line). Second, electric stimulation covers a wider frequency range than mechanical vibration in evoking somatic sensation (5–5,000 vs. 25–700 Hz), with electric stimulation being most sensitive at 20–50 Hz and vibration at 200–300 Hz (dashed black line). Third, because photons are particles, it makes no sense to compare between the visible light range (430–770 THz) and the 12–14 orders of magnitude lower electric stimulation frequency range (5–100 Hz). Instead, the electric visual frequency range is similar to the 2–64 Hz temporal frequency response range of retinal ganglion cells^49^. It is of interest to discuss similarities and differences between electric sensation and normal sensation at mechanistic levels.

### Mechanisms of electric sensation

To evoke sensation, transcranial electric stimulation must activate the sensory system at either the electrode-tissue interface, the receptor, the peripheral nerve, the central nerve, or a combination of all of the above. First, the electrode-tissue interface may convert electric stimulation into sound, vibration or light stimulation, which then evokes sensation normally. For example, low-level (1–10 μA) electric stimulation applied to a metal plate electrode on dry skin produces either auditory or tactile sensation by converting electric current into microvibrations similar to an electrostatic loudspeaker^11,50,51^. On the other extreme, high-level (10–30 mA) transcranial electric stimulation also produces sound, which is heard through bone conduction of vibrations in the entire skull^52^. Neither conversion mechanism is likely to account for the presently reported auditory or tactile sensation, which had thresholds in the 0.5–1.5 mA range, too high for the electrostatic vibration and too low for bone conduction.

Second, like normal stimulation, transcranial electric stimulation can activate the sensory receptors^8,12,53^. Although both types of photoreceptors, cones and rods, have been implicated in the sensation of electrically evoked phosphenes^6^, they cannot explain the maximal electric visual sensitivity at low frequencies (10–20 Hz). In contrast, the two types of cochlear hair cells may work together to account for the observed frequency range in auditory sensation, at least in normal-hearing subjects. The outer hair cells can be electrically activated by high-frequency electric stimulation^54^, whereas the inner hair cells by low-frequency electric stimulation^55–57^.

Third, transcranial electric stimulation may activate the peripheral nerve. In the retina, both bipolar and ganglion cells have intrinsic low-frequency electric resonance^58,59^, which would explain the maximal sensitivity of electric vision at low frequencies^60^. Electric current passing through the skin stimulates afferent nerve fibers, with thicker myelinated fibers being more easily stimulated than thinner unmylinated fibers, producing a causal relationship between the type of nerve activation and somatic sensation^13,61^. The presently observed dynamic range, 12-dB for tactile, 17-dB for visual and 12-dB for auditory sensation, is similar to the dynamric range in response to electric stimulation of the auditory nerve^62^ but much narrower than the 40-dB tactile, 70-dB visual and 120-dB auditory dynamic range in normal sensation^63^. The most convincing evidence for direct nerve activation is evoked visual sensation in blind subjects who lack photoreceptors^64^ and auditory sensation in the present two deaf subjects who lack cochlear hair cells. A computational model showing maximal stimulation of the auditory nerve with ear canal electrodes lend further support to the direct nerve activation hypothesis^46^.

Finally, direct activation of the central nerve is possible^65,66^ but unlikely to account for the present result. Similar to previous arguments against visual cortex activation^41,67,68^, the present study also found a significant electrode position effect: forehead electrodes (close to eyes) produced a higher incidence of visual sensation while ear canal electrodes produced a higher incidence of auditory sensation (Fig. 1d). Additionally, the location of sensation in the present study was always on the same side as the electrode position or symmetrical when a centered electrode on the forehead was used (e.g., Fig. 2). Had any cortex been directly stimulated, the location of sensation would be on the contralateral side^69–71^. Finally, the present transcranial electric stimulation delivered less than 1% current from the scalp to a deep target, namely the cochlea, in the head (Fig. 3f), which might be too low to activate any cortical neurons^69–71^.

### Limitations and implications

The present study was limited to characterizing sensation in response to short (500 ms with 300-ms steady-state) alternating-current stimulation. The present short duration was in contrast to much longer durations from seconds to tens of minutes in typical applications of transcranial electric stimulation^25–28^. The long duration in these typical applications was needed to induce or enhance clinically meaningful treatment effects but unlikely necessary for characterizing sensation in the present study. One reason for this unnecessity was that temporal integration is in the order of hundreds of milliseconds for visual, auditory and tactile sensation^72–74^. The other reason was adaptation, which reduces the sensation magnitude over time from seconds to minutes^75–77^. Although post-stimulus and carry-over effects have also been reported in both normal and electric stimulation^78–80^, the present study did not show any of these significant effects, possibly due to the short duration limitation. However, the short-duration limitation can be advantageous in event-related transcranial electric stimulation studies because short duration stimuli allow for temporal synchrony or modulation with 1–100 Hz brain oscillations^81–83^.

One important goal of transcranial electric stimulation is to deliver precise and targeted stimulation of deep neural tissues so that neural activities can be modulated to not only treat diseases but also enhance sensory and cognitive function. The present result showed that under the optimal electrode and stimulus configuration, namely inclusion of an ear canal electrode with high-current and low-frequency stimulation, transcranial electric stimulation could activate the auditory nerve inside the densest bone of the skull. Even under this optimal configuration, the gain was still less than 1%, suggesting that the overwhelming majority of current has dissipated to other tissues, causing side effects ranging from somatic sensation on the skin to undesirable activation of non-targeted neurons. The present low gain estimated with the cochlear implant is consistent with the result obtained with recordings taken in rodents and human cadaver brains^84^ or within the brains of people undergoing surgery for epilepsy^42^, further underscoring the need for understanding current flows and mechanisms of transcranial electric stimuilation in the human head.

Recently, simultaneous stimulation using multiple small electrodes and temporally interfering patterns has been proposed to improve gain, depth and focality of transcranial electric stimulation^85,86^. The present result suggests two additional approaches. One is to think beyond scalp-based electrodes by placing electrodes as close as possible to the target, for example, the present ear canal electrode stimulating the auditory nerve. It is conceivable to place a lens electrode in the eye, a brace electrode in the mouth, and a ring electrode in the nose to reduce distance or deliver novel electrode montages for targeted stimulation. The other approach is to identify and take advantage of intrinsic electric resonance properties of the targeted tissue^31^. For example, 10–20 Hz stimulation would most likely stimulate the retinal neurons^58,59^, while higher frequencies would stimulate the cochlear receptors^54–57^.

The present result may improve the design of human-machine interfaces for both normal and disabled people. For example, electric stimulation could not only produce clear speech and music sounds in normal-hearing individuals^9,87^ but also improve speech perception in hearing-impaired people^88^. In contrast to acoustic stimulation, auditory sensations evoked by electric stimulation are inaudible to others and can be resistant to noise. The present systematic characterization of electric sensation can also be used to avoid serious side effects as originally reported by Volta^1^, while helping restore normal sensation by either sensory substitution^89^ or complementing existing prosthetic devices^90^.

## Methods

### Subjects

**Figure 5:**
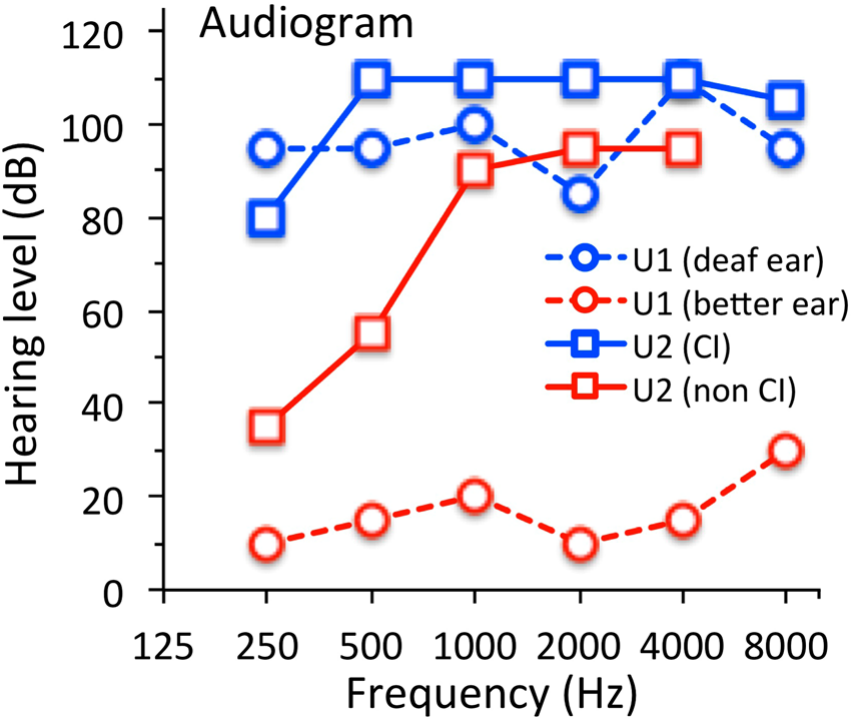
Audiogram for the two unilaterally deafened subjects. One subject, U1, was deaf in one ear (blue circles) and normal hearing (≤20 dB HL), except for mild hearing loss (30 dB) at 8,000 Hz, in the better ear (red circles). The other subject, U2, received a cochlear implant in the deaf ear (blue squares) and had residual low-frequency hearing in the non-implant ear (red squares).

Twenty human adults, aged between 20 and 67 years, participated in the study. All had age-appropriate hearing and vision, except for two subjects who had unilateral deafness. One of these two subjects received a cochlear implant on her deaf side. The cochlear implant (Nucleus5, Cochlear Ltd., Australia) had a built-in telemetry system, in which intra-cochlear electrodes could be used to record electrical impedance, electric potential, and neural responses in the cochlea^45^. Both unilaterally deafened subjects had severe-to-profound hearing loss (≥80 dB HL) in their deaf ears (blue squares and circles in Fig. 5) but either essentially normal hearing in the non-implant ear for one subject (red circles) or significant low-frequency hearing (35–55 dB HL) at 250 and 500 Hz in the better ear for the other subject (red squares). The University of California Irvine Institutional Research Board approved the protocol and methods in accordance with principles set forth in the Belmont Report and Declaration of Helsinki. All subjects received a written informed consent to participate in the study.

### Stimuli

**Figure 6:**
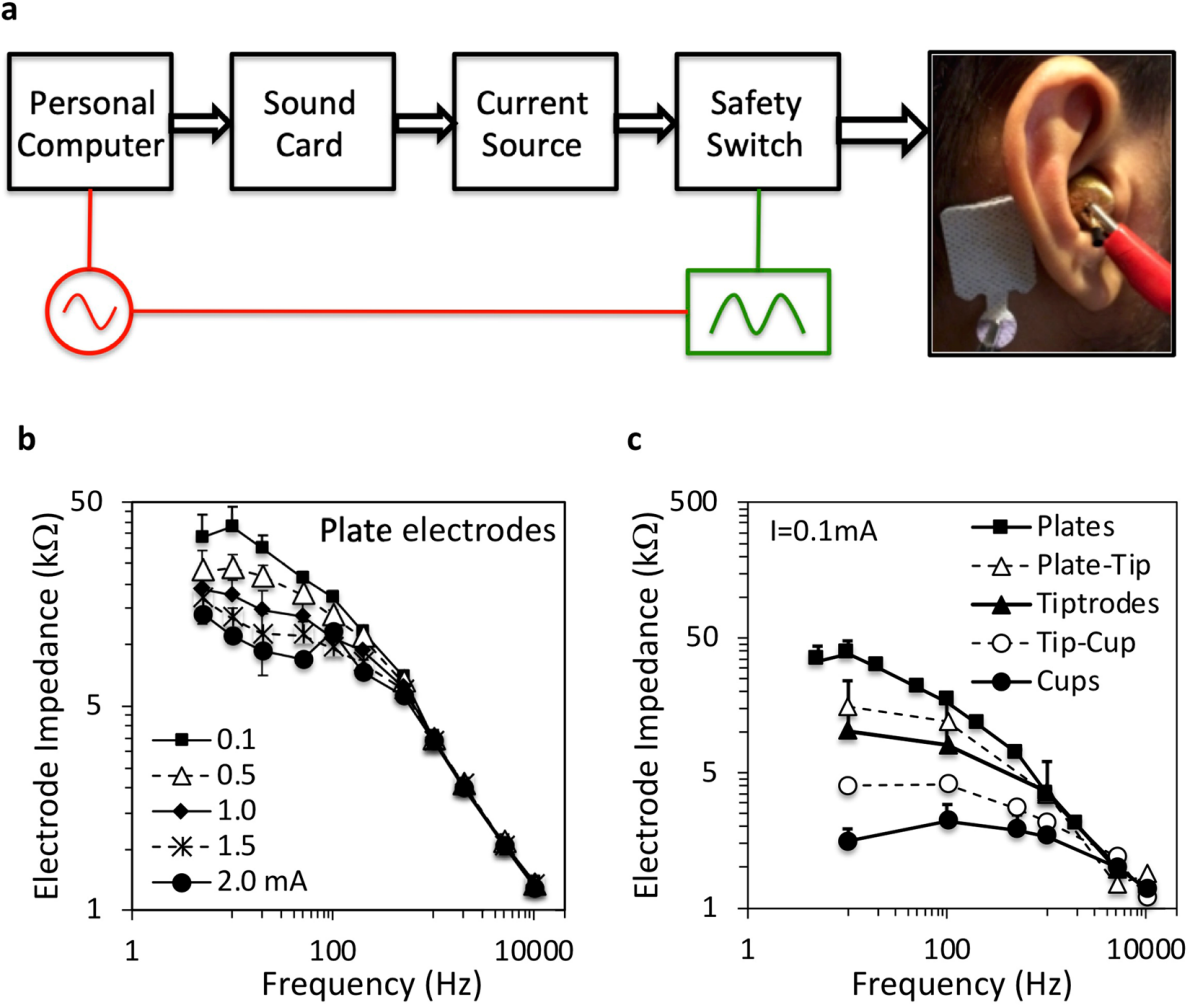
Transcranial electric stimulation setup and electrode impedance. (**a**) Block diagram of the step from personal computer to electrodes, showing a plate electrode on mastoid and a gold-plated tiptrode in the ear canal. The red circle represents a transformer power supply to isolate the power line from the subject and the green rectangle represents a monitoring oscilloscope. **(b)** Impedance for plate-to-plate electrodes as a function of stimulus frequency (x-axis) and stimulus level (symbols). **(c)** Impedance for different electrodes (symbols) as a function of stimulus frequency (x-axis) at a fixed 0.1-mA stimulus level.

Transcranial electric stimulation was delivered accurately and safely via a custom-built constant current source system (Fig. 6a). A personal computer generated digital stimuli and controlled timing of the stimulation. A sound card converted a digital signal into an analog signal. A current source converted the analog voltage stimulus into a current stimulus. A safety switch could turn off electric stimulation immediately by either the experimenter or the subject simply by releasing a foot pedal. Electric stimulation was delivered to a pair of electrodes placed on the subject’s head. A transformer power supply was used to isolate the subject from direct connection to mains. An oscilloscope was used to calibrate the equipment and to monitor the voltage delivered to the electrodes in real-time throughout the entire experiment. Before actual connection to the subject in each experiment, a 1,000-Ω resistor was connected to the output of the current source to calibrate the maximal output of the entire setup to be exactly 2 mA peak amplitude (or 4 mA peak-to-peak). Under no circumstance, would the current exceed 2 mA. The 1000-Ω resistor was disconnected during the actual test session.

All electric stimuli were charge-balanced, alternating-current sinusoids. The stimulus duration was 500 ms, including a linear 100-ms onset and offset ramp. The inter-stimulus interval was 1 second or longer depending upon the subject’s response time. The 11 stimulus frequencies included 5, 10, 20, 50, 100, 200, 500, 1,000, 2,000, 5,000 and 10,000 Hz. The low-frequency boundary of 5 Hz was chosen because frequencies lower than 5 Hz not only approached DC stimulation but also produced waveform distortion due to both current-source charge limitation and stimulus duration limitation (300-ms steady-state duration allowed barely 1.5 periods of 5-Hz stimulation). The high-frequency boundary of 10,000 Hz was chosen because previous studies showed little or no sensation caused by transcranial electric stimulation at higher frequencies^8,32,36,91^. The stimulus level was systematically varied from 0 to 2 mA but did not exceed a level at which any form of intolerable sensation was encountered. The default stimulus step size was 0.1 mA or smaller in case of a narrow dynamic range. No direct-current stimulation was used because the extended protocol (i.e., high level prolonged stimulation on small electrodes) might cause skin irritation^92^, hearing loss^93^ or other adverse effects^94,95^.

Electric stimulation was delivered to two electrodes, which could be a combination of the following 3 electrodes types, including (1) silver-chloride 2.3 × 3 cm plate (Natus Medical Inc., Pleasanton, CA), (2) gold-foil-wrapped-foam tiptrode (Etymotic ER3-26A, Elk Grove Village, IL), and (3) gold cup (Natus Neurology-Grass, Warwick, RI). The plate electrode could be placed on the forehead (Fpz), mastoid or temple. In addition to these 3 positions, the cup electrode could be placed on top of the head (Cz), with conductive gel (EC2 Electrode Cream, Natus Manufacturing Ltd., Gort, Ireland) being applied between the electrode and the scalp. The tiptrode or tip electrode was inserted only in the ear canal without any conductive gel. The maximum contact area was 6.6 cm^2^ (2.2 × 3 cm) for the rectangle plate electrode, 5.3 cm^2^ (r = 0.65 cm, height = 1.3 cm) for the cylinder tip electrode, and 0.8 cm^2^ (r = 0.5 cm) for the round cup electrode. The electrode impedance was systematically measured over both the stimulus frequency and level range for the plate-plate electrode configuration (Fig. 6b), showing a low-pass characteristic with nonlinear impedance below 1,000 Hz and linear impedance between 1,000 and 10,000 Hz for a general electrode-to-tissue interface^96,97^. The electrode impedance was also measured for 5 different electrode combinations at a fixed 0.1-mA stimulus level (Fig. 6c), and shows a direct relationship between impedance value and surface area when the electrodes were the same type (e.g., the plate-plate combination had the highest impedance), and an intermediate impedance value when the electrodes were different (the tip-cup combination had an impedance value between that for two tip electrodes and that for two cup electrodes).

A total of 10 electrode montages were used, including 4 involving the mastoid (to contralateral mastoid, contralateral temple, forehead and top), 4 involving the ear canal (to contralateral ear canal, contralateral mastoid, forehead and top), forehead to top, and temple to temple. The 10 electrode montages, combined with 2 electrode types, 11 stimulus frequencies, 20 current levels, and 20 subjects, produced ~88,000 independent observations of sensation in response to transcranial electric stimulation.

### Procedures

The experimenter first used an Electrode Skin Prep Pad (Dynarex Corp., Orangeburg, NY) to clean the target skin area of the subject. The experimenter then placed the proper electrode to the target skin area and secured the electrode or its connector and wire with tape to avoid electrode detachment or a loose connection. The experimenter examined the ear canal, and removed earwax if necessary, before inserting the tiptrode completely in the ear canal. The subject sat in a double-walled, sound-proof booth, with the light turned off. At the beginning of a test session, the experimenter made sure that the subject knew how to release the foot pedal to turn off electric stimulation in case of unpleasant or intolerable sensation. There were typically 4–6 test sessions with each session lasting for 3–4 hours. The subject could terminate the test at any time with or without cause.

The method of limits was used to determine sensation threshold, in which the stimulus level was increased step by step until the subject reported any type of sensation. The stimulus level was increased by several steps, then decreased step by step until disappearance of the sensation. The average of the two levels was reported as the threshold. In case of concomitant sensation, the threshold was first determined for the first sensation evoked by a lower stimulus level. The threshold was then determined for the second sensation evoked by a higher stimulus level, with the subject trying to ignore the first sensation as much as possible.

The method of magnitude estimate was used to determine the input-output function of the electrically evoked sensation. The subject was instructed to report a number between 0 and 10, with 0 being unnoticeable and 10 being unbearable, to describe the magnitude of the sensation at a suprathreshold stimulus level. The subject usually stopped the procedure when the sensation magnitude reached 8–9. The difference between the stimulus level at threshold and that at the 8–9 magnitude was defined as the dynamic range. Under no circumstance, did the stimulus level exceed 2 mA.

The method of adjustment was used to match the pitch of the electrically evoked auditory sensation to the frequency of a pure tone. The subject listened to the electric sound for as long and as often as needed, then adjusted the frequency of a pure tone to make its pitch much higher than the electric pitch as well as much lower than the electric pitch. The subject narrowed the frequency range until a frequency was reached to match the electric pitch.

Descriptive analysis on mean, standard deviation and histogram was used to characterize the threshold and input-output functions of human sensation caused by transcranial electric stimulation. A binomial distribution test statistic was used to determine whether two electrodes produced any significant difference in a sensation:

$$z=\frac{{p}_{1}-{p}_{2}}{\sqrt{p(1-p)(\frac{1}{{n}_{1}}+\frac{1}{{n}_{2}})}}$$

where *p*_1_ was the probability of a sensation caused by electrode 1, *p*_2_ the probability of the same sensation by electrode 2, *n*_1_ the total number of observations for electrode 1, *n*_2_ the total number of observations for electrode 2, and $p=\frac{{n}_{1}{p}_{1}+{n}_{2}{p}_{2}}{{n}_{1}+{n}_{2}}$. The null hypothesis (namely *H*_*o*_*: p*_1_ = *p*_2_) at the 95% confidence interval (*p* < 0.05) was rejected if *z*_*α/2*_ > 1.96 for the two-tailed test, indicating a difference in sensation between the two electrodes.
